# Emerging Roles of Gemin5: From snRNPs Assembly to Translation Control

**DOI:** 10.3390/ijms21113868

**Published:** 2020-05-29

**Authors:** Encarnacion Martinez-Salas, Azman Embarc-Buh, Rosario Francisco-Velilla

**Affiliations:** Centro de Biología Molecular Severo Ochoa, CSIC-UAM, Nicolás Cabrera 1, 28049 Madrid, Spain; azmane@cbm.csic.es (A.E.-B.); rfrancisco@cbm.csic.es (R.F.-V.)

**Keywords:** SMN, SMA, Gemin5, RNA-binding protein, translation control, reprogramming

## Abstract

RNA-binding proteins (RBPs) play a pivotal role in the lifespan of RNAs. The disfunction of RBPs is frequently the cause of cell disorders which are incompatible with life. Furthermore, the ordered assembly of RBPs and RNAs in ribonucleoprotein (RNP) particles determines the function of biological complexes, as illustrated by the survival of the motor neuron (SMN) complex. Defects in the SMN complex assembly causes spinal muscular atrophy (SMA), an infant invalidating disease. This multi-subunit chaperone controls the assembly of small nuclear ribonucleoproteins (snRNPs), which are the critical components of the splicing machinery. However, the functional and structural characterization of individual members of the SMN complex, such as SMN, Gemin3, and Gemin5, have accumulated evidence for the additional roles of these proteins, unveiling their participation in other RNA-mediated events. In particular, Gemin5 is a multidomain protein that comprises tryptophan-aspartic acid (WD) repeat motifs at the N-terminal region, a dimerization domain at the middle region, and a non-canonical RNA-binding domain at the C-terminal end of the protein. Beyond small nuclear RNA (snRNA) recognition, Gemin5 interacts with a selective group of mRNA targets in the cell environment and plays a key role in reprogramming translation depending on the RNA partner and the cellular conditions. Here, we review recent studies on the SMN complex, with emphasis on the individual components regarding their involvement in cellular processes critical for cell survival.

## 1. Introduction

Post-transcriptional mechanisms governing gene expression depend on the concerted action of RNA-binding proteins (RBPs) and RNAs. Soon after transcription commences, the mRNA associates with a distinct type of RBPs, giving rise to dynamic ribonucleoprotein (RNP) entities, which control gene expression as a function of the components present in the complex [1]. In this respect, RBPs play fundamental roles during the processing, modification, stability, localization and translation of RNAs [2,3]. Conversely, RNAs can regulate interaction, stability, localization, and function of RBPs.

Translation is the action of decoding mRNA information into protein. This is a tightly regulated process, predominantly controlled at the initiation step, which requires the activity of dynamic RNP complexes. Translation initiation in the vast majority of eukaryotic mRNAs occurs by a mechanism that depends on the m^7^GTP residue (or cap) located at the 5′end of most mRNAs. During cap-dependent initiation, eukaryotic initiation factors (eIFs) recruit the small ribosomal subunit to the 5′end of the mRNA [4]. However, there are alternative manners to initiate translation independently of the 5’cap of the mRNA. One of the best studied cap-independent mechanisms was characterized in viral mRNAs that evade cap-dependent inhibition and, at the same time, take advantage of the translation shutdown induced in infected cells [5,6,7]. This cap-independent mechanism is based on internal ribosome entry site (IRES) elements [8,9] which recruit the ribosomal subunits, promoting translation initiation at internal start codons [10,11]. Viral IRES-dependent translation requires a subset of eIFs and various RBPs [12,13,14], with the exception of the dicistrovirus intergenic region [15,16].

In every organism of all kingdoms of life, RBPs perform critical functions in cellular processes related to gene expression in general and more specifically, in RNA-driven mechanisms, affecting all steps of RNA life. Accordingly, RNA-dependent pathways can be altered by the reduced expression, aggregation, or sequestration of RBPs [17], finally resulting in disease. In addition, the malfunction of RBPs whose activity is directly involved in events related to translation control, including tRNA synthesis, ribosome biogenesis, sequestration, and mislocalization of mRNAs, causes a distinct type of disorders. Similarly, defects in proteins which determine the activity of macromolecular complexes can be the cause of severe diseases. For instance, the misregulated assembly of RNPs leads to neurodegenerative diseases such as amyotrophic lateral sclerosis (ALS) and spinal muscular atrophy (SMA). The latter is an autosomal recessive disorder caused by a reduction in the SMN (survival of motor neuron) protein [18], a central member of the SMN complex that governs the assembly of small nuclear ribonucleoproteins (snRNPs), the essential components of the spliceosome. Accordingly, the most frequent cause of SMA (about 95%) is due to mutations in the survival motor neuron 1 (*smn*1) gene, partially compensated by the *smn*2 gene. However, the activity of individual SMN proteins in each type of human cells and how the SMN complex contributes to motor neuron function and survival still remains unknown.

## 2. The SMN Complex Members and Functions

**Figure 1:**
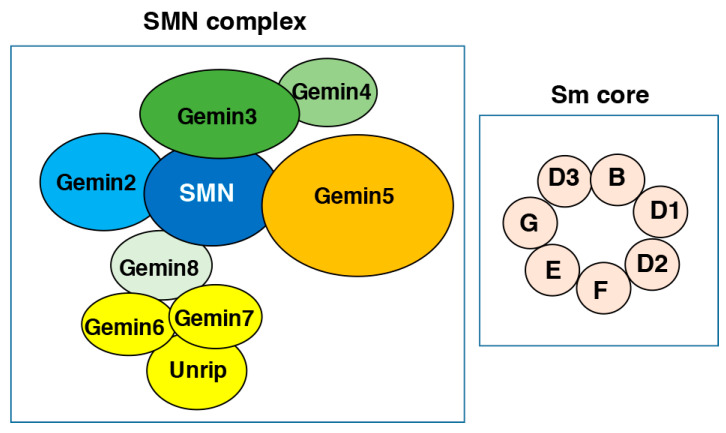
Simplified diagram of the survival of motor neuron (SMN) complex and the Sm core. The individual members of the SMN complex (SMN, Gemin2-8 and unrip) as well as the Sm proteins are depicted according to their interaction pattern. See text for details.

The SMN complex is expressed in all eukaryotic cells; it is present throughout the cytoplasm and in the nucleus, where it is concentrated in Gems (Gemini of Cajal bodies). In humans, the SMN complex comprises nine members (SMN, Gemins2–8 and the unr-interacting protein (unrip)) [19] (Figure 1). This complex is a molecular chaperone that plays a critical role in the biogenesis and function of snRNPs [20,21], the basic components of the splicing machinery. snRNPs consist of a U-rich small nuclear RNA (snRNA) and seven small (Sm) proteins that assemble onto the snRNA in the cytoplasm [22]. Two complexes, the protein arginine methyltransferase 5 (PRMT5) and the SMN, mediate the ordered assembly of the Sm proteins (D1/D2/F/E/G/D3/B) (Figure 1) through sequential steps. Newly synthesized Sm proteins remain bound to the ribosome near the polypeptide exit tunnel and dissociate upon association with the PRTM5 chaperone methylosome subunit pICln [23], which pre-arranges the Sm proteins into spatial positions. Then, the SMN complex dissociates pICln from the Sm proteins and enables their assembly on snRNAs to form snRNPs [24].

Sm ribonucleoprotein particles are ring-shaped structures formed by Sm proteins around a U-rich segment of RNA followed by a stem-loop. In Drosophila, SMN and Gemin2 proteins are sufficient to assemble the Sm core efficiently. The loss of SMN protein is lethal in humans, mice, and flies. A structural analysis of the SMN protein revealed a conserved oligomerization domain that contains a (YxxG)3 motif [25]. It is worth mentioning that about 50% of the missense mutations observed in SMA patients are located within the tyrosine-glycine (YG)-box of the SMN protein. Recent studies have shown that the oligomerization of SMN promotes protein stability by sequestering an Skp/Cullin/F-box(Slimb/ß) (SCF)^SLMB^ degron present in the YG-box [26]. Both the YG-box and the Gemin2-binding domain of the SMN protein are conserved between human and yeast. The human Gemin2 protein is monomeric, while the SMN-Gemin2 complexes form stable dimers which associate to form tetramers and octamers [27]. According to the estimated concentration of these proteins in living cells, it has been hypothesized that the dimers of SMN-Gemin2 are the most frequent form in the cell.

Gemin2 is the most conserved protein of the SMN complex and is responsible for the ordered assembly of SmD1/D2/F/E/G proteins into a pentamer prior to their gathering on snRNAs [28]. The modification of the phosphorylatable residue S81 of Gemin2 that lies between the motifs involved in Sm proteins binding [29] modulates the interaction with Gemin5, which also harbors several phosphorylation sites [30]. Gemin5 is responsible for the recognition of the U-rich sequence known as the Sm site of snRNAs through its tryptophane-aspartic acid (WD) domains and delivers these molecules to the SMN complex in the cytoplasm [31,32]. In addition to SMN, Gemin2, and Gemin5, the RNA helicase Gemin3 (DDX20) forms part of the minimal SMN complex mediating snRNP assembly in Drosophila [33]. The phosphorylation state of SMN, Gemin2, Gemin3, and Gemin5 determines their interaction. Gemin3 is a self-interacting phosphoprotein with RNA helicase activity controlled by the phosphorylation of S320, which mediates ATP binding [34].

Additional SMN complex members, such as Gemin4 that contains a nuclear localization signal, are essential proteins in mice. In human cells, the overexpression of Gemin4 induces the relocalization of the SMN complex to the nucleoplasm, while high levels of a Gemin4 variant lacking a nuclear localization signal sequesters Gemin3 and a fraction of Gemin2 in the cytoplasm [35]. Gemin6 and Gemin7 proteins exist as a heterodimer; they connect with each other via an interface similar to that which mediates interactions between Sm proteins [36]. Moreover, Gemin6, Gemin7, and Unrip form a stable cytoplasmic complex whose association with SMN requires Gemin8 [37].

The cellular levels of the individual members of the SMN complex are interconnected, such that low amounts of SMN protein, including those variants derived from SMA patients, decrease the stability of the other SMN complex components [38,39]. A reduction in SMN protein leads to a decrease in snRNP assembly, the disappearance of Gems, and a reduction in the amounts of several Gemins [40]. Conversely, a reduction in Gemin2 or Gemin6 strongly decreases the activity of the SMN complex. Given that Gemin2 makes multiple contacts within the SMN complex, an excess of Gemin2 results in the assembly of partial inactive complexes, reducing the amount of the functional complex.

The deletion of members of the SMN complex is incompatible with life. Low levels of SMN protein, as well as Gemins 2, 3, 4, 5, 8, or Unrip, is detrimental to cell survival in flies [41,42]. The disfunction of Gemin2, Gemin3, and Gemin5 results in defective motor phenotypes that are similar to those described for SMN in humans, while the loss of Gemin5/*rigor mortis* is lethal [43]. Furthermore, dominant-negative effects have been reported for defective SMN proteins unable to oligomerize, as well as for Gemin3 mutants lacking the helicase domain [44]. Conversely, the overexpression of Gemin2 has a toxic effect reducing flies’ viability, while the upregulation of SMN or Gemin5 impacts the viability of flies expressing a Gemin3 hippomorphic mutant [45]. Although the overexpression of Gemin5 protein in muscle tissues has no defects, flies exhibit viability disorders when coexpressed with the Gemin3 hippomorphic mutant. Taken together, all these findings highlight the relevance of the stoichiometry of the SMN members for SMN complex function.

The components of the SMN complex can be detected in the cytoplasm, nucleus, and nuclear gems. For instance, snRNP-cytoplasmic bodies (U bodies) contain the SMN protein and also Gemins 2, 3, and 5; U bodies are cytoplasmic granules associated with P bodies, which are involved in RNA surveillance and decay [46]. Regarding the implications of the cellular localization of the SMN complex, it has been hypothesized that the phosphorylation of its components can regulate cytoplasm-nucleus trafficking. Besides snRNP assembly and therefore spliceosome activity, regulatory signals affecting the phosphorylation of the SMN components can alter other activities of these proteins when acting individually. In support of these possibilities, SMN members have been identified as phosphoproteins in the cytoplasm of human cells [47]. A subsequent work reported additional phosphorylation sites in serine or threonine residues on SMN, Gemin2, Gemin3, Gemin4, Gemin5, and Gemin6, with SMN and Gemin3 showing the highest degree of phosphorylation [30].

Besides snRNP assembly, the SMN protein is involved in axonal mRNP assembly and trafficking [48]. In neurons, SMN can bind to mRNAs directly or via various RBPs; *Smn* cargos include mRNAs of β-actin, growth-associated protein 43 (*Gap43*), annexin 2 (*Anxa2*), and neuritin/*cpg15* [49,50,51]. Other functions reported for SMN protein include translation regulation [52], phosphatase and tensin-homolog-mediated (PTEN-mediated) protein synthesis pathways [53], neuromuscular junction function [54], and muscle architecture [55], reinforcing the biological relevance of this protein in distinct cellular processes. Furthermore, SMN also functions in the transport and assembly of microRNPs or telomerase RNPs [56,57].

## 3. The Modular Structure of Gemin5: Implications for Its Multifunctional Role in Gene Expression

Gemin5 is a predominantly cytoplasmic protein that forms part of the SMN complex in metazoan organisms [33,58,59]. The protein, which is expressed in all human tissues [60,61], is responsible for the recognition of the Sm site of snRNAs [32]. However, a large fraction of Gemin5 protein is found outside of the SMN complex [62], suggesting that it may have additional functions. In agreement with this view, Gemin5 acts as a hub for several RNP networks which perform a plethora of dedicated cellular functions. Among others, Gemin5 has been identified as a signal recognition particle (SRP)-interacting protein [63], as a ribosome-interacting factor [64,65], as a translation regulator of several mRNAs [66,67,68,69], or as a factor involved in trans-splicing [70]. Further supporting the involvement of this multifaceted protein in different activities, recent data have shown that Gemin5 can act as a reprogramming factor in zebrafish lateral line hair cells, allowing for a shift toward cell survival [71]. In addition, an RNA-Seq analysis revealed that the *gemin*5, together with *smn*1 and *gemin*3 genes, are linked to a common set of genetic pathways, including the tumor protein tp53 and the epidermal growth factor receptor ErbB pathways.

**Figure 2:**
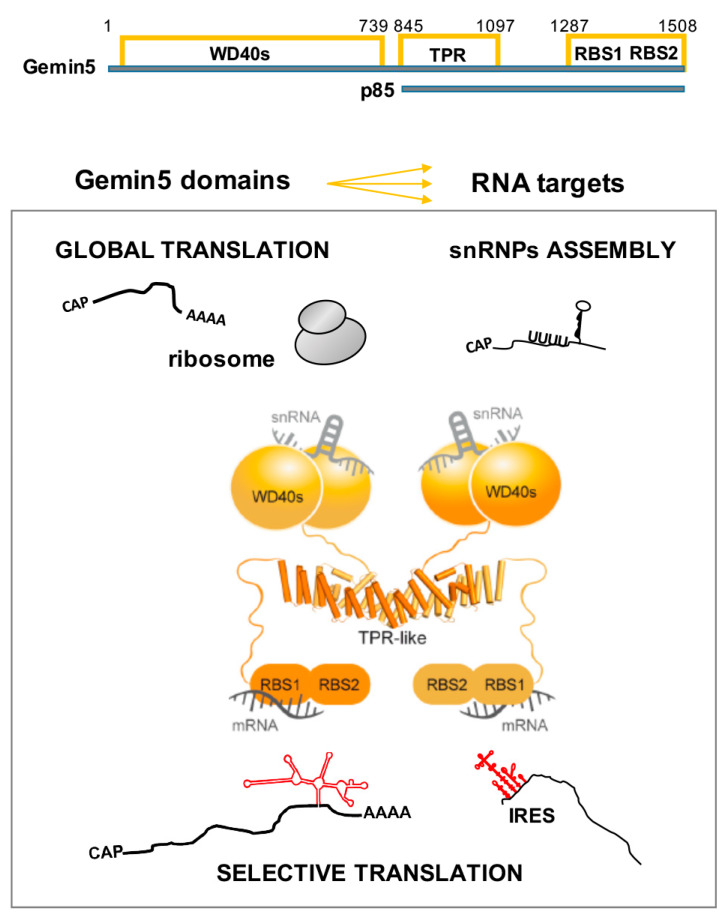
Schematic representation of Gemin5 domains and functions. The amino acids flanking each domain of the protein, as well as the p85 product resulting from the L protease cleavage, are indicated at the top. Known RNA targets of the different domains are depicted in relation to the observed function of the Gemin5 protein. The tetratricopeptide repeat (TPR)-like domain mediates the dimerization of the protein. See text for details on the impact of the indicated domains on the diverse functional roles of the protein.

The studies showing that Gemin5 acts as a downregulator of translation have underscored a new unexpected function for this protein [66], disclosing its contribution to RNA-dependent processes unrelated to the activity of the SMN complex. Then, the discovery that Gemin5 is a target of the L protease in picornavirus-infected cells [72], which produces a 85 kDa (p85) C-terminal polypeptide (Figure 2), has revealed important repercussions for the impact of the protein on the regulation of translation. Remarkably, the expression of p85 in human cells stimulates viral IRES-driven translation, contrary to the negative effect of Gemin5 in translation [73,74].

Gemin5 comprises distinct structural and functional domains (Figure 2). The N-terminal moiety (amino acids 1–739) is composed of two juxtaposed seven-bladed WD40 domains [75] that recognize the Sm site of snRNAs and the m^7^G cap via base-specific interactions [76,77]. The C-terminal part (amino acids 1287–1508) harbors a non-canonical RNA-binding site (RBS) with two moieties designated as RBS1 and RBS2 [73], which differ in RNA-binding capacity as well as in the ability to modulate translation. The RBS1 domain (amino acids 1287–1412) not only displays robust RNA-binding capacity in vitro, but also binds a selective group of RNA targets in living cells [68]. According to nuclear magnetic resonance (NMR) structural studies of the RBS1 polypeptide, this domain of Gemin5 does not adopt a unique structural conformation in solution [73], a feature frequently found in intrinsically unstructured proteins [78]. Therefore, separate modular regions of Gemin5 located at the protein ends with different structural organizations are involved in the recognition of RNAs performing distinct functions, such as assembly of snRNPs, ribosome interaction, and IRES-dependent translation.

More recently, structural analyses conducted to understand the functional role of the middle region of Gemin5 have demonstrated the presence of a robust dimerization module (amino acids 845–1097) (Figure 2) [74]. This singular dimerization domain, which resembles a canoe-shape, folds as a tetratricopeptide repeat (TPR)-like motif alternating 17 helical pairs that exhibit the classical TPR pattern of small and large hydrophobic residues [79,80]. Despite the tight association between Gemin5-TPR monomers, a single point mutation in the dyad axis is sufficient to destabilize the dimer [74]. The strong conservation of the TPR-like domain suggests that the dimerization module of Gemin5 plays a fundamental role in the architecture and activity of the protein. Moreover, the first residue detectable on the crystal structure coincides with the L protease cleavage site that releases the p85 fragment during foot-and-mouth disease virus (FMDV) infection [72], suggesting that the dimerization module plays a central structural role in the activities of this protein. In support of this possibility, a p85 construct with a tandem affinity purification (TAP)-tag at the C-terminus recruits the full-length cellular Gemin5 protein, while the mutant that prevents dimerization fails to do so. These data indicate that the canoe-shape domain provides the anchoring site for the interaction of p85 with the full-length Gemin5 protein in the cell environment, presumably contributing to the behavior of the viral-induced cleavage fragment in translation control. Among other possibilities, these results led us to propose that the recruitment of Gemin5 by the viral cleavage product p85 might reduce the pool of free full-length protein, thereby interfering with the RNA-mediated pathways where the involvement of this protein is required, including IRES-dependent translation. Consistent with this view, the disruption of the complex formation by the expression of the construct destabilizing the dimerization domain abolishes the translation-enhancing effect of p85 protein [74].

Beyond self-dimerization, the identification of p85-Gemin5-associated partners has disclosed relevant unknown functions of this versatile protein. The top gene ontology (GO) terms retrieved by p85 are splicing-related components, translation control members, and snRNP assembly factors, fully consistent with the functions reported for this protein [81]. Interestingly, a number of proteins associated with Gemin5 are involved in several unanticipated biological processes, such as the termination of RNA Pol II transcription, mRNA transport, cell-cell adhesion, or retina homeostasis, among others. Although these associations need to be characterized in detail, the data suggest that the protein can form part, either in a direct or indirect way, of multiple RNP networks. In marked contrast, GO terms associated uniquely with the mutant protein that fails to dimerize are related to apoptotic processes, proteolysis, and the response to unfolded proteins, strongly suggesting a protein instability problem caused by the destabilizing mutation of the dimerization domain.

Given that the dimerization mutant disrupts the p85-Gemin5 interaction, hampers the association with proteins regulating splicing and translation processes, and abolishes translation control, it is conceivable that defects in Gemin5 dimerization will occur in severe diseases, as shown in mutations affecting the TPR domains of other proteins [82]. Moreover, since Gemin5 appears to be connected with multiple cellular processes, it is plausible to think that defects in this protein can cause alterations in RNA metabolism, leading to cell disorders. However, the implication of Gemin5 protein in human disease induced by mutations causing diminished levels, sequestration, misfolding, or the inability to interact with relevant counterparts still remains to be elucidated.

## 4. The Gemin5 Interactome Unveils the Association with the Ribosome

The diverse composition of the cellular Gemin5 interactome reflects its functional versatility. A comprehensive view of the Gemin5 proteome reveals that most partners copurify with the long N-terminal domain [64], including several members of the SMN complex, RBPs, eIFs, and ribosomal proteins. Fully consistent with the interactome components identified, compelling evidence accumulated over the years supports the involvement of Gemin5 in gene expression and translation control pathways [66,67,83,84]. Nonetheless, many copurified factors are lost upon the exhaustive RNase A treatment of the complexes, uncovering the existence of Gemin5 networks guided by RNA bridges.

The association of the endogenous protein with ribosomal particles disentangles a novel feature of the Gemin5 protein [64]. Remarkably, the cellular protein sediments with the total ribosome fraction that contains ribosomal particles and eIFs. Furthermore, the purified protein interacts directly with 80S ribosomes and 60S ribosomal subunits through the N-terminal domain. Gemin5 and the G5_1-1287_ polypeptide as well are detected on heavy polysome fractions, reinforcing their ribosome-binding capacity (Figure 2) and also suggesting that the protein may have a partial stalling effect on translation elongation [64]. Consistent with this view, Gemin5 depletion or overexpression modifies the polysome profile, concomitant with an increase or a decrease, respectively, in global protein synthesis. Collectively, these data indicate that Gemin5 may control global protein synthesis via its binding with the ribosome.

In agreement with the Gemin5 capacity to bind 80S ribosomes, the ribosomal proteins associated with the N-terminal domain remain constant after RNase treatment. Moreover, the ribosomal proteins L3 and L4 interact directly with Gemin5 in glutathione-S-transferase (GST) pull-down assays, suggesting that Gemin5 can form a binary complex with these proteins. L3 and L4 are conserved proteins with terminal extensions located on the solvent side of the ribosome [85,86,87]. L4 forms part of the peptide exit tunnel wall [88], while L3 coordinates the binding of elongation factors and aminoacylated tRNAs. These data are compatible with the hypothesis that Gemin5 interaction with L3 and/or L4 can cause the observed negative effect on global translation. In this context, the increase in the polysome/80S ratio observed after the overexpression of Gemin5 may be indicative of the preferential translation of selective mRNAs concomitant to the decrease in the elongation rate of the majority of mRNAs, as previously reported [89]. Thus, the ribosome binding capacity of the N-terminal moiety enables Gemin5 to control global protein synthesis, while the C-terminal end harbors the repressor element of IRES-dependent translation [73]. Hence, this protein contributes to translation control in different ways using its distal domains, which additionally have the capacity to recognize specific partners in the cell environment [64].

## 5. Promoting and Repressing Roles of Gemin5 in Translation Control

In recent years, Gemin5 has emerged as a critical factor during translational reprogramming events, although the mechanisms underlying this phenomenon are still poorly known [71]. In this respect, previous studies have shown that Gemin5 regulates translation through its capacity to recognize a selective subset of mRNAs [68] and also serves as a hub for numerous RBPs [64] that, in turn, are involved in gene expression control. This protein-RNA adaptor model can help to explain its role in translational reprogramming. As mentioned before, Gemin5 acts as a negative regulator of IRES-dependent translation [66,90], but it also acts as a translation stimulator for the SMN mRNA [67]. Noteworthy, the RNAs used in the studies mentioned above are dissimilar in several respects, including the different location on the mRNA of the motif recognized by Gemin5 (5´UTR, or 3´UTR, respectively) as well as main differences in the secondary structure of the putative RNA motif. In this regard, the predicted secondary structure of 3´UTR of the SMN mRNA appears to resemble the snRNAs secondary structure recognized by the N-terminal end of Gemin5 [32]. In contrast, the protein binds to the IRES element within a conserved stem-loop followed by a single-stranded region via the non-canonical RNA-binding site placed at the C-terminal end [91].

The marked differences in RNA recognition reported by different studies are compatible with the capacity of the individual domains of Gemin5 to recognize still-unknown RNAs. This possibility prompted the search for cellular RNA targets of the RBS1 domain using a modified crosslinking and immunoprecipitation (CLIP)-based approach in HEK293 cells. A prominent feature of the RNA hits specifically recognized by the RBS1 domain is the ability to fold as thermodynamically stable hairpins [68] in full agreement with the RNA-binding properties of the C-terminal domain of Gemin5. Remarkably, none of the RBS1-associated sequences belong to snRNAs, indicating that the RBS1 region of Gemin5 specifically recognizes a different set of RNAs than the WD-repeats domain [31,32]. Regardless of the expression of the RBS1 domain individually, the Gemin5 CLIP data deposited in ENCODE (https://www.encodeproject.org) shows a 30% overlap with RBS1 hits, further supporting the RNA-binding capacity of RBS1. These studies identified an internal region of the Gemin5 mRNA, designated H12 for hits 1-2, among the RNA targets of both Gemin5 and RBS1. The biochemical and functional characterization of this target has demonstrated that the RBS1 polypeptide physically interacts with the H12 RNA region upregulating mRNA translation, thereby providing a regulatory feedback loop that counteracts the negative effect of Gemin5 on translation control. Further supporting this feedback loop, the expression of RBS1 alone stimulates the translation of the endogenous Gemin5 mRNA [68]. In addition, RBS1 stimulates the translation of mRNAs harboring the H12 sequence independently of its position on the mRNA. These results provide support for the relevance of the RBS1 moiety in translation control and demonstrate a regulatory mechanism that depends on both the mRNA target sequence and the expression of the RBS1 domain.

In summary, Gemin5 could contribute to translation control in different ways using distinct functional domains and their respective RNA partners (Figure 2). The negative role in the global translation of the N-terminal region of Gemin5 is related with the capacity to recognize the cap structure of RNAs, to bind the ribosome, and to sediment with heavy polysomes [84]. Instead, the negative effect of RBS1 on translation is due to the enhancing translation effect on the endogenous Gemin5 mRNA. Consequently, an increase in the amount of Gemin5 protein downregulates the global protein synthesis.

Besides the different functions of Gemin5 in normal conditions, the possibility that this protein can have an important impact on translation control under cellular stress is still an open question. As mentioned before, Gemin5 was identified as a factor bound to two viral IRES elements—hepatitis C (HCV) and FMDV [92]. Moreover, Gemin5 silencing stimulates FMDV and HCV IRES activity [66] in tissue culture cells. It is well established that IRES-dependent translation initiation mechanisms drive the translation of specific mRNAs under conditions that impair cap-dependent translation initiation as well as in certain stages of development, differentiation, and cell proliferation [93,94,95,96]. Furthermore, IRES-dependent translation is regulated by RNA-interacting proteins among others by Gemin5 [97,98,99,100]. Under normal conditions, Gemin5 negatively regulates IRES-mediated translation initiation, a function achieved by the RBS2 domain located at the most C-terminal end [73]. However, as mentioned before, Gemin5 is cleaved in FMDV-infected cells by the L protease [72], such that the resulting p85 C-terminal polypeptide enhances IRES-driven translation, therefore reprogramming viral gene expression. In this respect, an additional example of the enhancing effect of Gemin5 in translation is the IRES element reported in the thioredoxin-interacting protein (TXNIP) mRNA [69].

Regarding the role of Gemin5 in the regulation of gene expression under severe stress as it occurs during viral infections, it has been shown that the protein is redistributed to the viral factories during Sindbis virus (SINV) infection co-localizing with SINV RNA [101]. Indeed, the overexpression of Gemin5 inhibits SINV capsid proteins synthesis. In agreement with the role of Gemin5 in cap-dependent translation [64], Gemin5 recognizes the 50 nt located at the 5´ end of the genomic gRNA and subgenomic RNA (sgRNA) of SINV, which contain a cap structure at their 5´ends. In addition, Gemin5 binds to the downstream loop a long hairpin that stimulates the translation of the sgRNA [102], preventing SINV RNA translation and therefore impairing SINV infection. Currently, whether Gemin5 performs a critical role in other virus infections remains an open question.

## 6. Conclusions

RBPs participate in many steps of the mRNA lifespan and are frequently associated in large macromolecular complexes. However, the role of individual RBPs in the expression of a given RNA in living organisms and how RBPs cooperate to form networks are still poorly understood. The works reviewed here recapitulate the involvement of the SMN members in various cellular processes, including snRNP assembly and translation control. Numerous studies have shown that RBPs with functions related to neuromuscular diseases, such as the components of the SMN complex, display a modular structure, allowing their interaction with different partners presumably in response to specific cellular signals. Beyond its RNA-binding activity in the SMN complex, Gemin5 cooperates in translation control and ribosome binding through separate structural and functional domains of the protein [81]. Collectively, these observations strongly suggest that Gemin5 can form part of distinct functional RNP complexes which could be involved in biological processes relevant to cell survival. In this respect, many questions remain open regarding the functions performed by the Gemin5 protein individually. First, the biophysical properties of the protein in the cytoplasm remain to be studied. Second, the identification of pathogenic events downstream of Gemin5 mutations needs to be elucidated. Hence, it is very likely that the extent of Gemin5 involvement in disease, as well as that of other components of the SMN complex, will grow in the next years.

## Abbreviations

**ALS**	amyotrophic lateral sclerosis
**eIFs**	eukaryotic initiation factors
**FMDV**	foot-and-mouth disease virus
**Gems**	*gemini* of coiled bodies
**HCV**	hepatitis C virus
**IRES**	internal ribosome entry site
**pICln**	Methylosome subunit pICln
**PRMT5**	protein arginine methyltransferase 5
**RBPs**	RNA-binding proteins
**RBS**	RNA-binding site
**RNPs**	ribonucleoprotein complexes
**SINV**	Sindbis virus
**Sm**	7 small proteins
**SMA**	spinal muscular atrophy
**SMN**	survival of motor neuron
**snRNAs**	small nuclear RNAs
**snRNPs**	small nuclear ribonucleoproteins
**TPR**	tetratricopeptide repeat motif
**unrip**	unr-interacting protein
**UTR**	untranslated region
**WD**	tryptophan-aspartic acid motif
**YG-box**	tyrosine-glycine motif
